# A Changeable Lab-on-a-Chip Detector for Marine Nonindigenous Microorganisms in Ship’s Ballast Water

**DOI:** 10.3390/mi9010020

**Published:** 2018-01-05

**Authors:** Myint Myint Maw, Xinxiang Pan, Zhen Peng, Yanjuan Wang, Long Zhao, Bowen Dai, Junsheng Wang

**Affiliations:** 1 College of Marine Engineering, Dalian Maritime University, Dalian 116026, China; parahitamyint@dlmu.edu.cn (M.M.M.); panxx@dlmu.edu.cn (X.P.); 2 College of Information and Science Technology, Dalian Maritime University, Dalian 116026, China; pengzhen@dlmu.edu.cn (Z.P.); wangyanjuan@dlmu.edu.cn (Y.W.); zl_swl@dlmu.edu.cn (L.Z.); dbw1120160153@dlmu.edu.cn (B.D.)

**Keywords:** microalgae, bacteria, microfluidic chip, resistance pulse sensor, fluorescence detection

## Abstract

The spread and invasion of many nonindigenous species in the ship’s ballast water around the world has been a hazard and threat to ecology, economy, and human health. The rapid and accurate detection of marine invasive species in ship’s ballast water is essential. This article is aimed at analysing ballast water quality by means of a changeable microfluidic chip detector thus comply with the D-2 standard of ship’s ballast water management and sediment convention. The detection system was designed through the integration of microfluidic chip technology, the impedance pulse sensing and LED light induced chlorophyll fluorescence (LED-LICF) detection. This system can measure the number, size, shape, and volume of targeted microorganisms, and it can also determine the chlorophyll fluorescence intensity, which is an important factor in analysing the activity of phytoplankton. The targeted samples were *Chlorella volutis*, *Dunaliella salina*, *Platymonas subcordiformis*, *Chrysophytes*, *Escherichia coli*, and *Enterococci*. The whole detection or operation can be accomplished through online detection in a few minutes with using micron volume of the sample solution. The valid data outputs are simultaneously displayed in terms of both impedance pulse amplitudes and fluorescent intensity signals. The detection system is designed for multi-sizes real time detection through changing the microchannel sizes on the microfluidic chip. Because it can successfully detect the label-free microorganisms, the system can be applicable to in-situ detections with some modifications to the system.

## 1. Introduction

With the development of the world shipping industry, the hazard and threat of marine invasive species to the marine ecology, economy, environment, and human health has become extremely significant and serious [1]. The supply of ship’s ballast water and the draining process is the most common cause of the spread of marine non-indigenous organisms in the sea. These have already caused the catastrophic damage to the marine ecosystems. It also causes fouling of ships, buoys, and harbour structures [2]. Ship’s ballast water generally contains various species of microalgae, bacteria, viruses, etc. More research needs to be conducted on methods to prevent the introduction of unwanted species via ballast water. Recognizing the possible severity of the consequences, the International Maritime Organization (IMO) has taken action by adopting the “International Convention on the Management of Ships Ballast Water and Sediments” (hereafter will be used as convention) [3]. The convention entered into force on 8 September 2017 [4], and ships must have an on-board a ballast water management (BWM) plan approved, ballast water record book and an international ballast water management certificate. Existing ships, constructed before 8 September 2017 are required to comply with either ballast water exchange Standard D-1 or ballast water performance Standard D-2 while all new ships must meet Standard D-2 after it entered into force [5]. This article will focus on the application of Standard D-2 performance. Ships need to install approved ballast water management systems, which will ensure that the discharge water meets the defined performance standard with strict limits on concentrations of viable organisms and microbes. The limitations are as follows: the discharge limitation of equal or larger size than 50 µm organisms must be less than 10 viable in one cubic meter; the limit size of discharging for less than 50 µm organisms and larger or equal to 10 µm organisms shall be lower than 10 viable organisms in millilitre; the limit concentration of indicator microbes for *Escherichia coli* is set at 250 cfu (colony forming unit) per 100 mL, *Enterococci* is 100 cfu per 100 mL and toxicogenic vibrio cholera is set at 1 cfu per 100 mL [6].

The ship’s ballast water-borne organisms contain a huge number of phytoplankton and zooplankton, in which the phytoplankton mainly are microalgae. Therefore, the detection of microalgae and bacteria in the ship’s ballast water not only analyzes the related quality of ballast water, but also is aimed at balancing the ecological environment and economy interests of each country.

Historically, microalgae and bacteria are detected by microscopes, commercial Coulter counter machines, image analyzers and flow cytometry. By using a microscope, the particle size can be measured and the number of particles in the sample can also be counted. This is the classical method and benchmark method for biologists [7,8,9]. There are some obvious shortcomings in this approach. Microorganisms can be affected by temperature, light intensity, pressure value during the transit of samples, which may cause some changes to samples’ characteristic.

The Coulter counting method generates a pulse when a particle passes through a small hole between two electrodes that are suspended in the electrolytic solution [10]. There are commercially available Coulter counting equipment [9,11,12,13], and nowadays it is widely used in ocean engineering. When compared with the microscope, the Coulter counting device is bulky and expensive. In the image analyzing method, a high quality camera is essential and is combined with a microscope [9,14,15]. To develop the image information of cell morphology, the image analysis algorithm needs more study. The method of flow cytometry is quite popular in detecting fluorescence in samples [9,16,17,18]. However, this can also be done only in the laboratory due to the large size and complex software processing.

Based on the conventional methods of analysis, the use and results are quite reliable for analyzing microorganisms. The challenges in applying those conventional methods of analysis in the marine sector lie in the collection of marine microbial samples. The characteristics of the sample may be affected by changing environment during its transportation to the laboratory and at the laboratory. The detection time also takes longer and hence the results do not come out immediately. Therefore, a portable online detection device enabling fast response portable for detecting marine microorganisms is essential.

In the age of sophisticated technology, microfluidic chip devices have increasingly attracted the scientific community attention [19,20,21]. Microfluidic technologies are evaluated as one of the 15 most important inventions for the future of mankind. They have many advantages in technology, such as the need of few samples and reagent consumption, speedier detection, high efficiency in analysis, and the miniaturization of volume. They are easy to fabrication and integrate and are also portable. They are widely used in the fields of biology, medicine, chemistry, and environment [22,23,24,25,26]. In the last few years, microfluidic impedance pulse sensing, which is also known as the resistance pulse sensing (RPS) has been developed [27,28,29,30] and even used in the detection of nano particles. The combination of microfluidic chip and the impedance pulse sensing method can count individual microorganisms and determine their sizes, while the combination of microfluidic chip and light induced fluorescence method can analyze the viability of fluorescent cells. In this paper, taking their characteristics into account and in order to eliminate the limitations of the existing methods, we choose the option to design the detection system by combining the microfluidic chip technology, the impedance pulse sensing method, and the light induced chlorophyll fluorescence technique to detect and analyze the targeted microorganisms. Microalgae were targeted as sample of phytoplankton; and *Escherichia Coli* and *Enterococci* were chosen as samples of indicator bacteria for detection system.

## 2. Materials

### 2.1. Overall Design of Detection System

**Figure 1:**
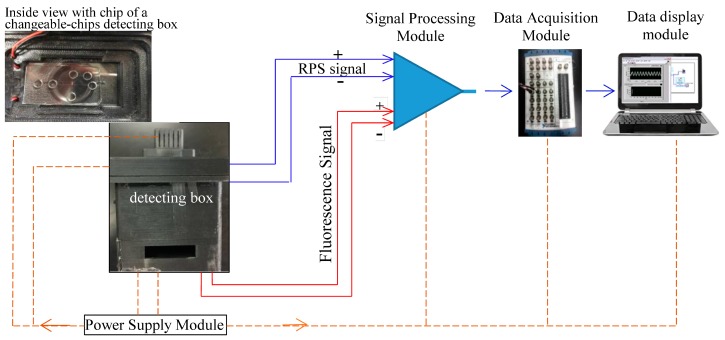
The principle diagram of overall design of detection system.

The detection system consists of a changeable-chip detecting box, signal detecting module, signal processing module, data acquisition module, power supply module, and also the display data and analytical module (see Figure 1). The chip box is a substrate of microfluidic chip. The detection module includes impedance pulse sensing sub-module and LED light induced chlorophyll fluorescence detection sub-module. The signal processing module includes preamplifier circuits, filter amplifier circuits, and signal processing circuits. The data acquisition module receives digital signal results. The power module supplies to impedance pulse sensing, electrode holder, and to four electrodes, LED light excitation, fluorescence detector, photodiode, signal processing circuits, and twenty four volt DC regulated power the whole detection system. Finally the data display module includes display screen and analyzing Lab VIEW software (2011 version, National Instruments, Austin, TX, USA).

### 2.2. Design of a Microfluidic Chip

**Figure 2:**
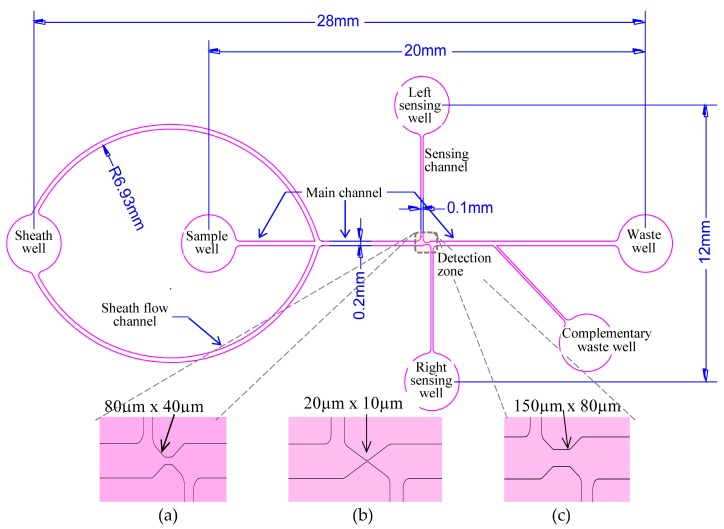
Diagram of microfluidic chip design and dimension (above) and detail dimension of various detection zones; (**a**) 80 µm (L) × 40 µm (W) × 40 µm (H) for 3 μm–20 µm size cells or particles (**b**) 20 µm (L) × 10 µm (W) × 8 µm (H) for 500 nm~2 µm cells or particles (**c**) 150 µm (L) × 80 µm (W) × 80 µm (H) for 20 μm–50 µm organisms cells or particles.

A polydimethylsiloxane (PDMS) microfluidic chip was the platform of the whole detection system. A detailed design of the microfluidic chip and its dimension are shown in Figure 2 (above). The microfluidic chip consists of a sample well, a sheath well, left and right sensing wells, a main waste well, a complementary waste well, oval shaped sheath channel, main straight channel, detection zone, two sensing channels which are laterally perpendicular to main channel and a 45-degree inclined waste channel. The total length between the sheath well and waste well is 28 mm. The distance between two centers of left- and right- sensing wells is 12 mm. All of the wells are of the same size with 3 mm–4.5 mm diameter and 2.8 mm–3.5 mm height. The width of the sheath channels can be generally larger than other channels to induce a hydrodynamic effect to the samples, and their dimension is between 200 mm and 500 mm. Using one sheath well assists in the flow of phosphate-buffered saline (PBS) solution with equal pressure distribution into two sheath channels. The main channel size is designed so that it is at least four times of the width of the detecting zone. The design basic parameters of detection zone are very important factors in detecting living microorganisms. Furthermore, a thirty degree inclination from the main channel to the detection zone allows the sample flow equally in its individual channels. We have proposed three optimal dimensions for detecting zones: 150 µm (Length, L) × 80 µm (Width, W) × 80 µm (Height, H); 80 µm (L) × 40 µm (W) × 40 µm (H); 20 µm (L) × 10 µm (W) × 8 µm (H); according to the results from several experiments (details data are not mentioned here) for detecting between 20 μm–50 µm size of microalgae, and also for between 3 μm–20 µm size of microalgae, and, for bacteria, 1 μm particle and until 2 μm microalgae. These three optimal dimensions of detection zones are illustrated in Figure 2a–c (below). The complementary waste well and its channel are added into the system by inclining 45° from main channel. This ensures a consistent flow of sample solution in the main channel. Its channel size is the same as the size is of sensing channels. The detection microorganisms can be fully completed on a quite small microfluidic chip by supporting the small sample volume without external aids.

### 2.3. Fabrication of the Microfluidic Chip

The chip was fabricated by bonding a PDMS layer and a glass substrate through applying the soft lithography technology. At first, AutoCAD designs of microfluidic chip were used to print out the photomasks. Following the standard protocol of soft lithography technology, the negative photoresist of SU-8 2025, 2005, or 2035 (Micro Chem Co., Newton, MA, USA) was layered on a cleaned and well baked 4 inches silicon wafer circular disc (Lijing Co., Ltd., Quzhou, China) by using a spin coater (G3P-8, Cookson Electronics Equipment, Indianapolis, IN, USA) at the speed of 1640 rpm, 1000 rpm, 1200 rpm, respectively. Then, the photomask was mounted on the silicon wafer disc. This was excited by UV from OAI Model 30 UV light source and OAI 150 UV exposure timer (Optical Associates Inc., San Jose, CA 95134 USA). After rinsing disc by using SU 8 developer, we got the master mold that can be used in manufacturing the desired quantities of PDMS microfluidic chip. To get the PDMS, we used 10 times weight in grams of silicone elastomer to a weight of the curing agent (Sylgard 184, Dow Corning Corporation, Midland, MI, USA). The mixture was degassed for about 3 h, and then it was poured onto the substrate and baked for 5 h in the oven under 80 °C temperature (Isotemp vaccum oven model 280A, Fisher Scientific, Pittburgh, PA). The baked PDMS was cured and formed as the same design for the master mold. The duplicate PDMS was peeled from the master mold, it was cut as a desired pattern, was punched for wells, and cleaned for 40 s together with a glass slide (50 mm × 24 mm × 0.15 mm, Citotest Labware Manaufacturing Co., Ltd., Haimen, China) in plasma cleaner (PDC-30G, Harrick Plasma, Ithaca, NY, USA). The PDMS surface was bonded with the channels and the glass sheet and gave the complete fabricated PDMS microfluidic chip.

### 2.4. Fundamental Materials for LED Light Induced Fluorescence Detection

Cell’s size and the pigment compositions of microalgae are the focus in our experiments to analyze the cells’ activity. Therefore, the rest experiments are also based on detection chlorophyll fluorescence spectral changes in microalgae samples. We proposed the LED light induced chlorophyll fluorescence (LED-LICF) detection system. To set it up, we choose the LED bulb (LZ1-10B200, central wavelength of 485 nm, LED Engin Inc., San Jose, CA, USA), as the excitation source to the samples solution with a LED driver (STCS2ASPR, STMICROELECTRONICS, Geneva, Switzerland), a collimator, a plano-convex lens (23.9 mm diameter, 12 mm thickness, and, 14 mm focal length), 480 nm bandpass filter (ET480/40 m, passing central wavelength 480 nm with FWHM 40 nm bandwidth, Chroma Technology Corporation, Bellows Falls, VT, USA), and a biconvex lens (12.7 mm diameter, 4.6 mm thickness and 13 mm focal length). We placed 685 nm emission bandpass optical filter (NBPF685/30, passing central wavelength of 685 nm and FWHM of 30 nm, Shanghai Mega-9 Optoelectronic Co., Ltd., Shanghai, China), and a silicon photodiode (PD) (S8745-01, Hamamatsu, Bridgewater, NJ, USA) under the microfluidic chip drawer to receive the emission chlorophyll fluorescence light with a feedback of resistor and capacitor.

### 2.5. Mechanical Structure of a Detection Box

**Figure 3:**
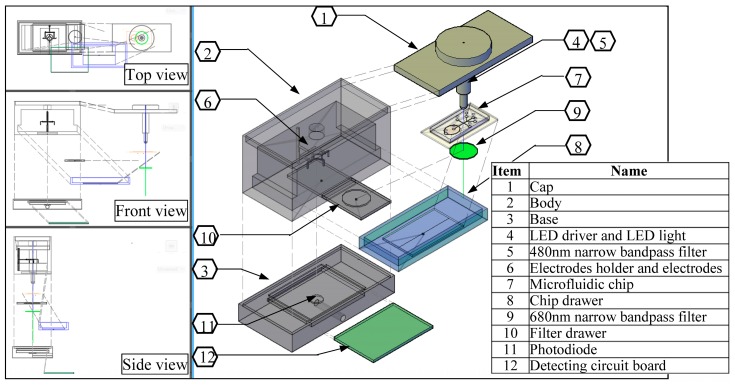
The assembly drawing of the detection box.

The detail assembly drawing of the detection box is as shown in Figure 3. It basically consists of 12 items: cap; body; base; light excitation source; optical filter; electrodes stand; microfluidic chip; and, chip platform drawer, fluorescence emission bandpass filter, filter drawer, photodiode, and power circuit board. The outside structure dimension is 100 mm (L) × 54 mm (W) × 72 mm (H) × 5 mm thickness. The purpose was to prevent the light entering from the surrounding the whole structure was made with black color plastic-fiber. The electrode stand supports the vertical movement of the electrode holder up and down in a groove. The minimum position of it will ensure that all of the electrodes are submerged in each well, while the maximum position will make them far from the microfluidic chip. The microfluidic chip’s drawer and optical filter’s drawer were designed to be pushed manually in and out. This is easy to operate in changing various channel design of microfluidic chips, and also the emission optical filter can be changed anytime. The most important component of the system is such that the excitation LED light and microchip detection zone, center of filter and the photodiode must be in line. The detecting fluorescence signal circuit and the sensing impedance pulse circuit are set at the base of structure.

### 2.6. Sample Preparations

The targeted samples of the detection system are microalgae and bacteria. Four types of microalgae were chosen as samples in this study. On the other hand, the indicated bacteria by Standard D-2 of Convention, *Escherichia coli*, and *Enterococci* were taken as samples in this experiment.

#### 2.6.1. Samples Solution of Microalgae

Four different kinds of microalgae: (1) *Chlorella volutis* (*C. volutis*); (2) *Dunaliella salina* (*D. salina*); (3) *Platymonas subcordiformis* (*P. subcordiformis*); and, (4) *Chrysophyeae* (*Chrysophytes*) samples were obtained from Marine Bioengineering Society, Dalian Institute of Chemical Physics, Chinese Academy of Sciences, Dalian, China. Their sizes, shapes, and colors are not the same. *C. volutis* are spherical in shaped 2 µm to 8 µm in diameter and contains chlorophyll-a and -b. *D. salina* are rod to oval in shaped 10 μm to 12 µm cell sizes and green algae. *P. subcordiformis* are about 12 μm to 21 µm cell size and contain chloroplast. *Chrysophytes* are cylindrical, vase, or funnel in shape, 4 μm to 10 µm. They were all cultured in enriched seawater medium with the certain amount of trace element solution, minerals, and vitamins at room temperature. The microalgae were healthy and as near as possible to the same condition as in their natural environment. Other contaminants and undesired oversize of microalgae were removed by corresponding fine filters from each microalgae cultured solution. Each 2 mL of filtered solution were deconcentrated with 20 mL of deionization water, and then prepared as sample solutions by combining 2 µL of deconcentrated microalgae, and 1 mL of phosphate buffer saline (PBS) solution, which 1:10 mixed up PBS and deionization water.

#### 2.6.2. Sample Solution of Bacteria

*Escherichia coli* (*E. coli*) (ATCC 25922) and Enterococcus faecalis (ATCC 29212) in petri dish with Luria-Brentani (LB) were provided by Jie Su et al., Marine Ecology Department, National Marine Environmental Monitoring Centre, Dalian, China. To prepare the sample bacteria, *E. coli* isolated from the petri dish was put into 5 mL of LB broth (pH 7.0) test tube, while Enterococcus isolate was put into the 5 mL of BHI broth test tube and cultured overnight at 37 °C incubator with 130 rpm speed. Each bacteria solution was centrifuged and diluted ten times with deionization water. 5 µL of each diluted bacteria solution was cultured with 1 mL of treated and fine filtered natural sea water with salinity 32. Then, bacteria condition was checked under inverted microscope (Nikon Eclipse Ti-E, Nikon, Japan). The dimensions of both bacteria were measured: *E. coli* has in common 560 nm–720 nm in diameter and 1 μm–2 µm in length; and enterococcus has 650 nm–800 nm in diameter and 1μm–1.5 µm in length.

## 3. Methods

### 3.1. Detection Principle of Impedance Pulse Sensing

Impedance pulse sensing, also known as resistive pulse sensing (RPS), is a method for counting and sizing cells or particles that will increase in impedance when passing through a small orifice or a small channel suspended in an electrolytic solution [31,32]. In the detecting system, the sample well is connected with a positive electrode and the waste well is connected with a negative electrode. When 24 V DC power is supplied between the sample well (*V_+_*) and the waste well (*V_−_*), the main channel becomes the equivalent to three resistors as *R_s_*, *R_d_*, and *R_w_*. Among them, *R_d_* is located at the resistance at detection zone, *R_s_* and *R_w_* are resistances at main channel of sample well side and waste well side. The circuit produces the increased impedance *R_d_* in real time when the targeted sample passes the detecting zone, and it was converted into the voltage pulse. The changes at *R_d_* can be calculated by Equation (1), because Deblois and Bean used Maxwell’s approximation theory to evaluate the resistance of the cylindrical channel containing a spherical particle [33],

(1) $$\Delta R=\frac{4\rho d^{3}}{\pi D^{4}}\times F\left(\frac{d^{3}}{D^{3}}\right)\left(\mathrm{for}\;d\ll D\;and\;\frac{D}{L}\ll 1\right)$$

where *ρ* is the resistivity of the suspending solution, *d* is the diameter of particle, *D* and *L* are the diameter and the length of the detection zone for a cylindrical shape, $F\left(\frac{d^{3}}{D^{3}}\right)$ is a correction term for non-uniformity of the current density. As for Equation (1), the change of impedance is inversely related to the fourth power of channel’s diameter rather than positively correlated with the size of detected particle. It means that the increasing the length and diameter of the detecting zone will decrease the sensitivity and also reduce the detection limit. Therefore, in the case of detecting multi-sizes samples, the channel dimensions of the detection zone must be considered.

Theoretically and practically, the change in resistance results the change in voltage [34]. According to our microfluidic chip detection system design, the changes in resistance *R_d_* at detecting zone will cause the changes of potential difference at both ends of sensing channels, *V_left_* and *V_right_*. This two voltage changes when the sample particles passing through the detection zone can be calculated with Equations (2) and (3) [35].

(2) $$\Delta V_{left}=-\frac{R_{s}\Delta R}{{\left(R_{s}+R_{d}+R_{w}\right)}^{2}+\left(R_{s}+R_{d+}R_{w}\right)\Delta R}\left(V_{+}-V_{-}\right)$$

(3) $$\Delta V_{right}=\frac{R_{w}\Delta R}{{\left(R_{s}+R_{d}+R_{w}\right)}^{2}+\left(R_{s}+R_{d+}R_{w}\right)\Delta R}\left(V_{+}-V_{-}\right)$$

Also, the two electrodes from sensing wells are supplied with power via the detecting impedance pulse circuit. It produces very weak output voltages, and, therefore, these are processed with preamplifier circuit to amplify the signal outputs, with filter amplifier circuit to filter noises from signals and amplify the signals until they can be interpreted. When the signal processing circuit receives the change of the output voltage, it can be calculated by the following Equation (4).

(4) $$\Delta V_{output}=A\left[\left(\eta_{left}-\eta_{right}\right)+\left(\frac{R_{d}(V_{+}-V_{-})}{R_{s}+R_{d}+R_{w}}\right)+\left(\Delta V_{left}-\Delta V_{right}\right)\right]$$

where *A* is the voltage gain by the filter amplifier, *η_left_* is the noise of the channel between the sample well and the detection zone, *η_right_* is the noise of the channel between the waste well and the detection zone.

Since the two channels are relatively close, the noise signals difference is very small. So, the formula of the first polynomial value can be approximately equal to 0 and also the formula in the second polynomial value can be seen as constant. Then, the change in value mainly depends on the last polynomial term. That is, the potential difference between the two ends of the detecting area can be expressed by Equation (5).

(5) $$\Delta V_{output}=-A\frac{\left(R_{s}+R_{w}\right)\Delta R\left(V_{+}-V_{-}\right)}{{\left(R_{s}+R_{d}+R_{w}\right)}^{2}+\left(R_{s}+R_{d+}R_{w}\right)\Delta R}$$

It is seen that the value of Δ*V_output_* is dependent on the value of Δ*R* in the Equation (5) and the value of Δ*R*, the amount of increased resistance is positively related to the size of samples in Equation (1). Therefore, we can summarize that: when the sizes of samples are larger, the resistances are bigger, and hence the amplitude of voltage signal outputs will be longer.

### 3.2. LED Light Induced Chlorophyll Fluorescence (LED-LICF) Detection Principle

Light induced fluorescence technology is a mature detection technology [36,37]. There are many advantages of using LEDs, such as being less expensive, consuming less energy, more stable, and allowing for a greater range of wavelengths. Therefore, the LED light induced chlorophyll fluorescence (LED-LICF) method that delivers light energy to the subsurface and records the fluorescence spectral output was applied to the system [38,39,40]. The main characteristics of microalgae to be considered are their sizes, pigment composition, chloroplast structure, cell wall, flagellum number, and the location of living environment [41]. In fact, microalgae metabolism, photosynthesis, energy utilization, and other physiological characteristics are related to its size as well, as the pigment composition. Likewise chlorophyll plays an important role in the photosynthesis of microalgae [42]. The content of chlorophyll in microalgae can determine the activity of microalgae.

**Figure 4:**
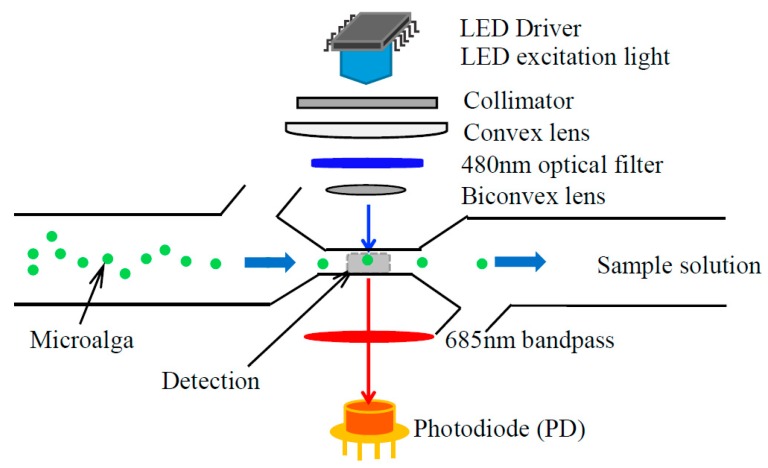
Diagram of LED light induced chlorophyll fluorescence detection principle.

The detection principle of LED-LICF is shown in Figure 4. The blue-light beam emitted by LED light source passed through the collimator, Plano convex lens, blue light filter, and biconvex lens to the detection zone of the microfluidic chip. The samples solution will flow through the detection zone along the main channel from the samples well to the waste well under the action of hydraulic pressure. When the samples cells, particularly microalgae, are excited by LED light, the corresponding chlorophyll fluorescence (generally red fluorescence) of microalgae are emitted due to energy loss and so the frequency of the excitation light becomes lower. They are received by the silicon photodiode (PD) after passing the red bandpass filter. The chlorophyll fluorescence signals were converted into the voltage signals by signal processing circuit. The size of the voltage signal represents the content of chlorophyll of the microalgae cell, and it also indirectly provides the information of activity and the type of microalgae cell.

### 3.3. Working Procedures

Firstly all of the modules were set up, as shown in Figure 1 in Section 2.1.

Secondly, once the samples were available, 30 µL of sample solution was pipetted into the sample well of a microfluidic chip, while PBS solutions with a less amount of sample solution was introduced into the sheath well, two sensing wells, and the waste well. A complementary waste well was not loaded with any solution. Because the solution levels were different between the samples well and both waste wells, a hydraulic pressure was created on the system. It let the sample solution flow from the sample well. Then, the loaded solutions microfluidic chip was carefully placed in the chip’s drawer. It was checked to see whether the center of detection area of chip was in line with the center of drawer. Then chip drawer was put inside the structure. After that, the electrode holder was moved down until the minimum position, which ensured that each electrode was embedded into the correspondence wells.

Finally, 24 V DC power regulator was switched on to supply the samples well electrode and the waste well electrode, and also to the sensing impedance circuit, the detection fluorescence circuit, and the LED excitation light source circuit. When the power was supplied, the detecting system automatically operated and the output results for resistance pulse sensing (RPS) and fluorescence signals were displayed on the computer screen by the aids of LabVIEW software.

## 4. Results and Discussions

Since the system was fully assembled, it was necessary to verify whether all of the modules in the system met the requirements of detection during its operation. The system was tested with standard polystyrene fluorescence microparticles (particles A–E with five different diameters between 4 μm–20 µm, Millipore SIGMA, Sigma-Aldrich Inc., Germany) to ensure the reliability and stability of the system until the relevant experiments’ results were verified. First, we separately tested the reliability of system for impedance pulse changes and tested later for fluorescence intensity (all data were not shown here). We applied Equations (1) and (5) to calculate the specific sample size from the amplitude of RPS signal result. We also calculated the ratios of each Δ*V_output_* for five different particles A-E with Equation (5) and their relations become as Equation (6) in ideal case.

(6) $$\Delta V_{output)A}:\Delta V_{output)B}:\Delta V_{output)C}:\Delta V_{output)D}:\Delta V_{output)E}=d_{A}^{3}:d_{B}^{3}:d_{C}^{3}:d_{D}^{3}:d_{E}^{3}$$

By comparing the results from Equations (1), (5), and (6), the system can be decided whether there are valid conditions for impedance sensing. The fluorescent particles produced green fluorescence under the radiation of a blue LED light excitation source and the photoelectric detector (PD) received green fluorescence as signals via a green emission bandpass filter. Here, the detection system was allowed to change not only the microfluidic chips, but also the designated emission bandpass optical filters. After that, we checked the system’s reliability through the integration of impedance pulse sensor (RPS) and together with a LED light induced fluorescence detector. This means the system could simultaneously receive RPS signals related to the size of sample particles and the correspondence fluorescence signals. All of the detection results were transmitted through DAQ board to the installed LabVIEW software host computer and displayed on PC.

**Figure 5:**
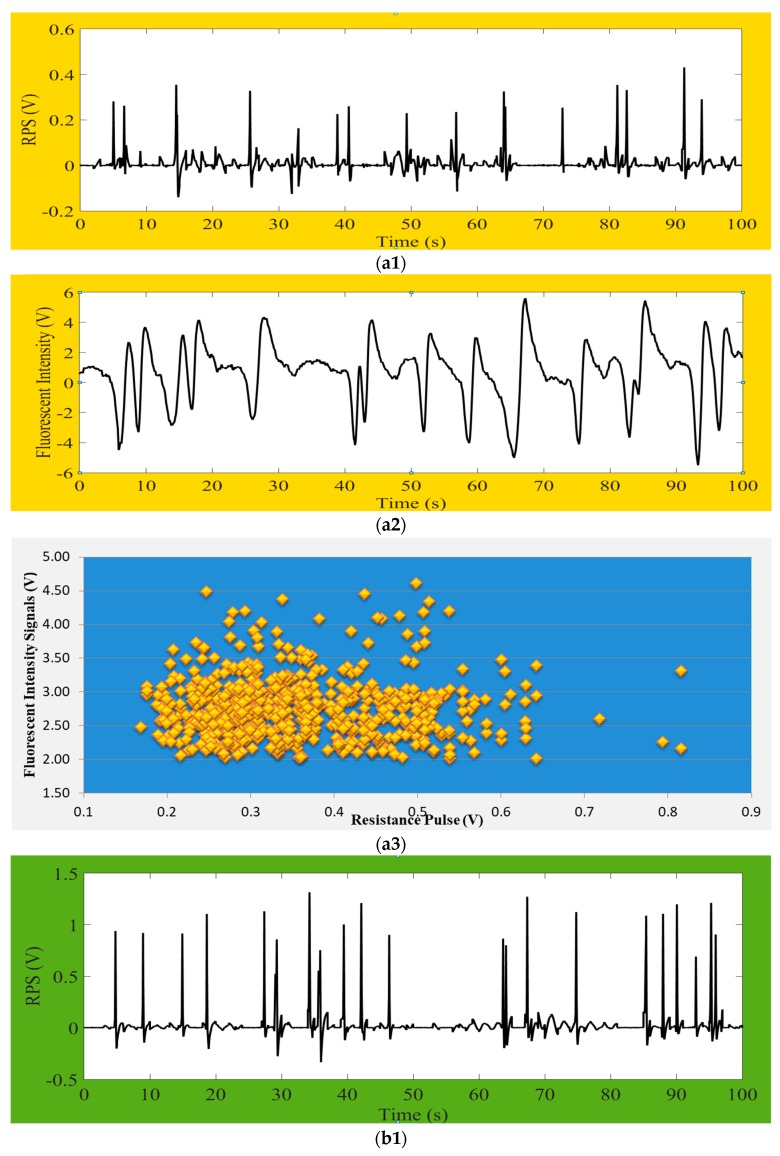

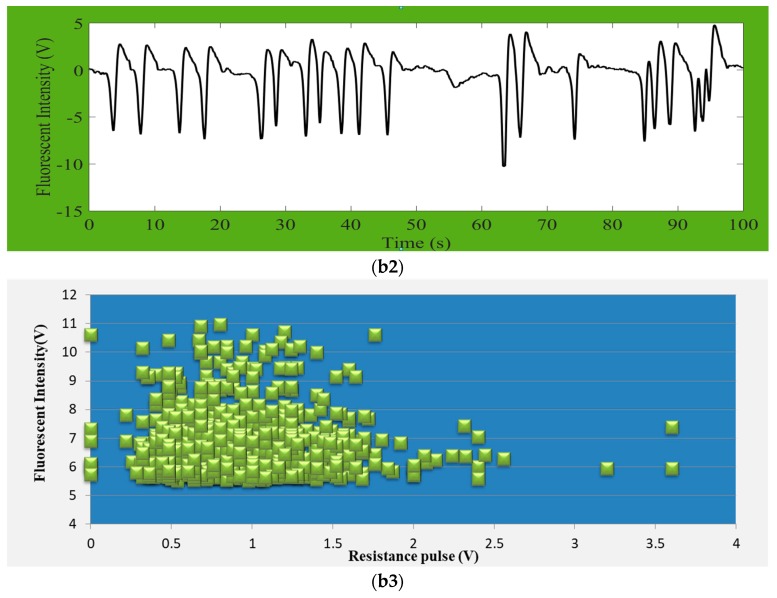
Diagrams of (**a1**) impedance pulses-; (**a2**) chlorophyll fluorescence signals- and (**a3**) total detected cells- for about 5 µm size of *Chrysophytes* microalgae and; (**b1**) impedance pulses-; (**b2**) chlorophyll fluorescence signals- and (**b3**) total detected cells- for about 10 µm size of *D. salina* microalgae

Once the detection system was ensured for the validity, following the above detection procedures and detecting principles, the average size of 20 µm *P. subcordiformis* microalgae samples were detected by using a detecting zone of 150 µm (L) × 100 µm (W) × 100 µm (H) microfluidic chip. The results of both impedance pulse signals (RPS pulses) and fluorescence signals were displayed on a computer screen (the data was not shown here). Similarly, other microalgae samples, consisting of *Chrysophytes* microalgae of about 5 µm size and *D. salina* about 10 µm size, were detected by providing a detecting zone of 80 µm (L) × 40 µm (W) × 40 µm (H) microfluidic chip. Their correspondence result signals of impedance pulses were shown in Figure 5(a1,b1), fluorescence signals were shown in Figure 5(a2,b2), and also total quantities of detected microalgae in during process were shown in Figure 5(a3,b3). In fact, the variations of microalgae sizes and their chlorophyll content cannot be controlled, so the signals amplitude and intensity amplitudes causes changes in the result figures. Fluorescence intensity is being produced based on the particle chlorophyll content, and so each intensity signal will be aligned with correspondence RPS signal. Our detection system found no misaligned events. Nevertheless, there will have no fluorescence intensity if the microalga is dead. The figures clearly indicate that the value of RPS is larger when the microalgae size is larger. We received the RPS amplitude range from 0.2 V to 0.8 V for *Chrysophytes* microalgae and the RPS amplitude range between 0.45 V and 3.5 V for *D. salina*.

In the Figure 5(a1,a2,b1,b2), y-coordinate represents the signal amplitude of impedance pulses (RPS) or chlorophyll fluorescence intensity and x-coordinate shows online real detection time. The amplitude of the fluorescence signal represents the activity of microalgae. It could distinctly be seen that each fluorescence signal from microalgae had correspondence with each impedance pulse signal, but every single RPS signals do not have to produce fluorescence signals. The reason for this is that the detected fluorescence represents the chlorophyll content in microalgae cell, and there will have no chlorophyll when the microalgae cell is dead but the size and volume of cell can be detected by RPS. Besides, the amount of fluorescence intensity positively correlates with the chlorophyll content of microalgae cells (this means that the higher the chlorophyll content, the stronger the microalgae fluorescence intensity would be under the certain excitation of light). It also shows their characteristic. Therefore, it is possible to determine whether the detected particles are microalgae active or not by means of chlorophyll fluorescence. It can also classify the microalgae according to the different chlorophyll content. By comparing these two microalgae’s results of impedance pulse signals and fluorescence signals, we can summarize that the larger the volume of microalgae, the greater the amplitude of the generated signal, and as long as the microalgae flows through the detection area it will produce the corresponding pulse signal. The impedance pulse sensing technology is used for counting and volume measurements of particles.

**Figure 6:**
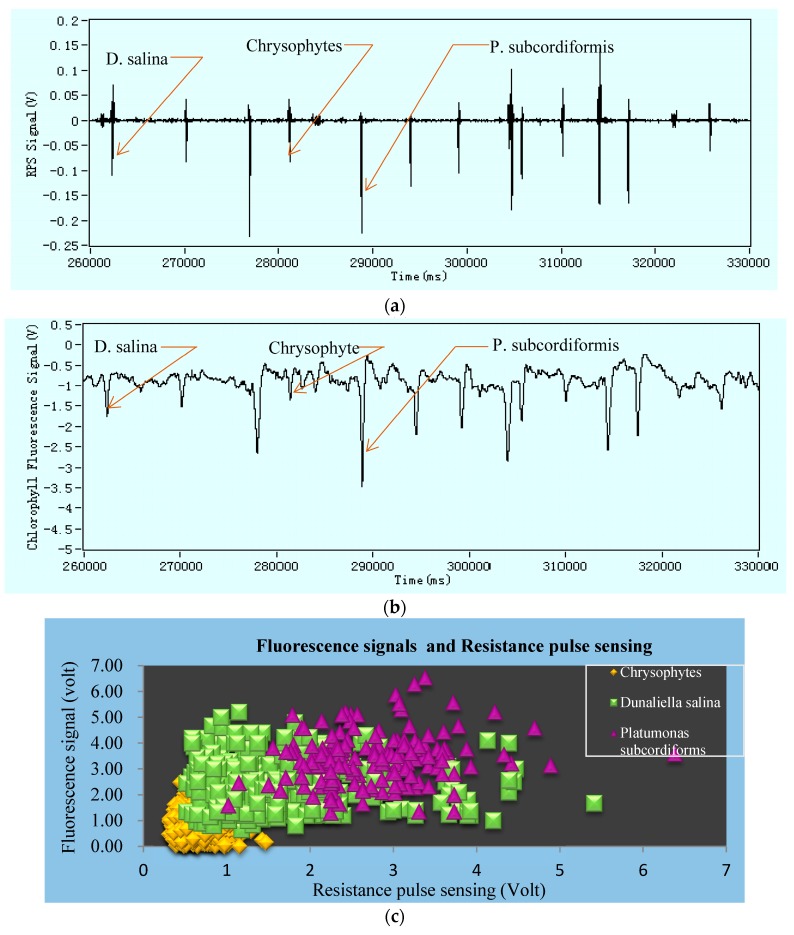

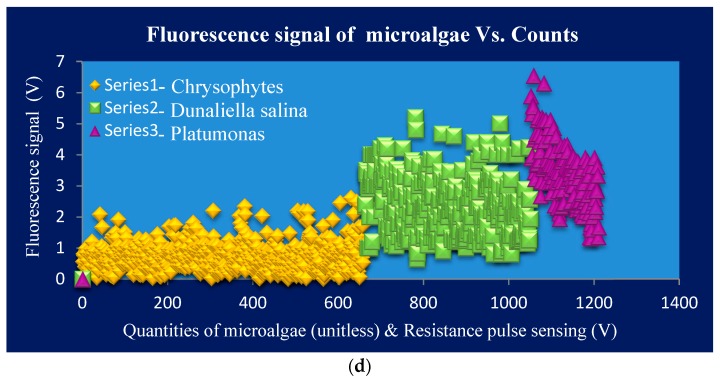
Diagrams of (**a**) only impedance pulse signals and (**b**) only chlorophyll fluorescence signals (**c**) the results regarding fluorescence and resistance pulse sensing (RPS) (**d**) total counts in certain duration for simultaneous detection of *Chrysophytes*, *D. salina* and *P. subcordiformis* microalgae.

On the one hand, the actual ship’s ballast water may contain various kinds of microorganisms. The simultaneous detection of different large sizes on a same chip becomes a challenge for system stability and validity. This issue was solved by grouping the range of microorganisms’ sizes. Firstly, each 1 µL of three microalgae samples solution: *Chrysophytes*; *D. salina*; and, *P. subcordiformis*; were mixed in a test tube and added PBS buffer solution for culturing. It was noted that the mixed samples solution was not centrifuged. The results output of specific impedance pulse signals and specific fluorescence signals of mixed sample solutions were shown in Figure 6a,b. The positive correlation of sizes & their RPS, the correspondent fluorescent intensity in microalgae, and the detected quantities for one complete detection are shown in Figure 6c,d. The types of samples on simultaneous detection in the RPS system can be distinguished by comparing the values of RPS signals amplitude and their volumes. So, the Equation (6) can be applied for the calculation. However, microorganisms’ characteristics are very complex and their sizes undefined; we proposed each record detection results for individual microorganisms on the same type of microfluidic chip. These results will provide to judge in distinguishing specific type of microorganisms.

According to the Figure 6, the detection system still follows the correlation rules that the larger the volume or size, the higher the RPS and then the stronger the fluorescence intensity will be when the chlorophyll content is higher. Also, the detection zone dimension does not conflict with the size range and is still stabilized in a higher signal-to-noise ratio.

**Figure 7:**
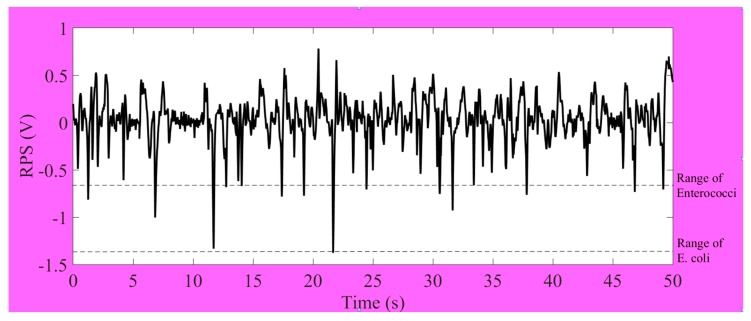
Diagram of the range of impedance pulses for simultaneous detection *Enterococci* and *E. coli*.

The minimum and maximum detection limits of a microfluidic chip can be determined on the basis of the dimensions of the detection zone. The comparative sizes of the detection zone in relation to particle size determine the accuracy of the systems operation. Too large a detection zone in relation to particle size will lead to insignificant signals or no signals at all. In our project, bacteria size is rather smaller than the microalgae cell and so the generated signals are very weak. According to the detection limit of microfluidic chip, the dimension of the detection zone had to be optimized to get the higher signal-to-noise (S/N). Label-free *E. coli* and *Enterococci* bacteria were simultaneously detected by 20 µm (L) × 10 µm (W) × 8 µm (H) detecting area of microfluidic chip. The output results of impedance pulse signals are shown in Figure 7. Generally, the actual sizes of specific bacteria are different. In regard to the original sizes of bacteria, their detection ranges were different. We generally got the range of RPS signals of *Enterococci* between 0.2 V and 0.65 V. The range of RPS signals of *E. coli* is between 0.5 V and 1.4 V. It can be claimed to distinguish for the overlapping amplitudes of RPS in our system. The specific detection results chart and signals profiles allow for us to distinguish the type of bacteria present in the sample.

**Figure 8:**
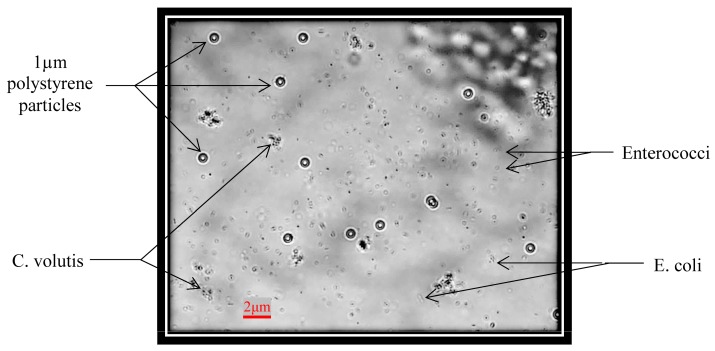
The optical image of *Enteroccoi*, *E. coli*, 1 µm polystyrene particle and *C. volutis*.

**Figure 9:**
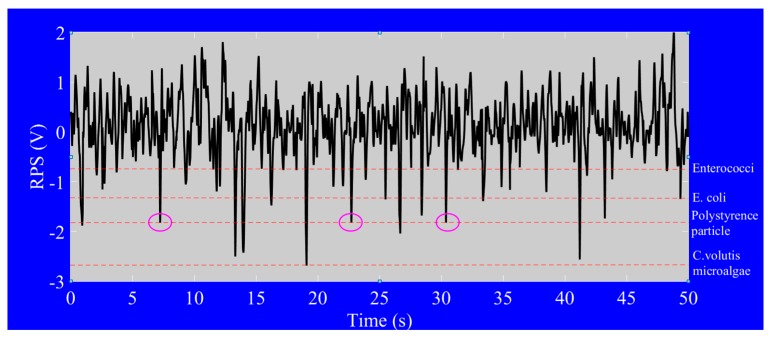
The diagram of impedance pulse signals (RPS) of *Enterococci*, *E. coli*, 1 µm standard particle and *C. volutis*.

**Figure 10:**
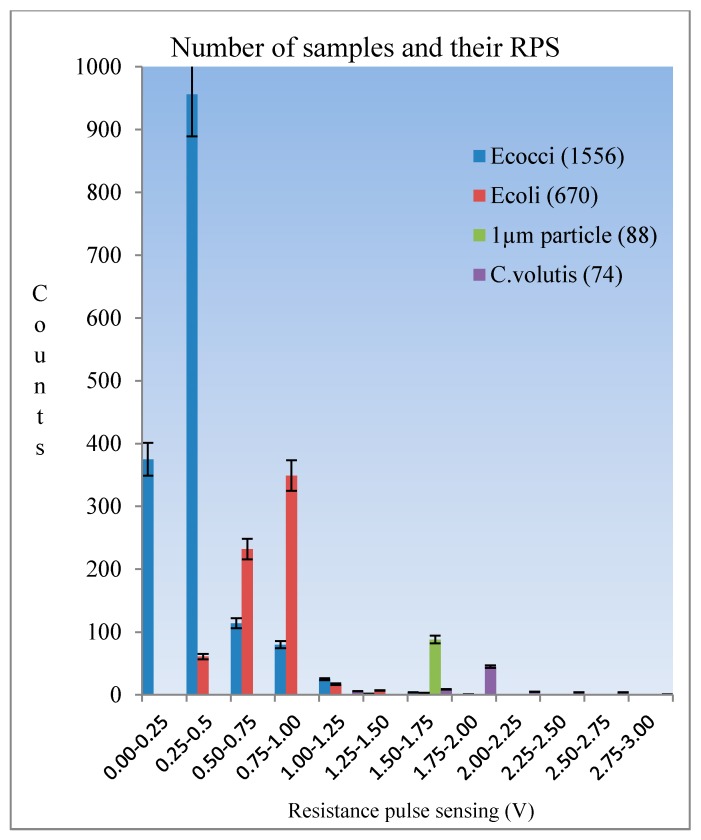
The statistic RPS diagram for total detected numbers of *Enterococci*, *E. coli*, 1 µm standard particle and *C. volutis*.

Furthermore, there may be some sediment, such as non-living things, in real ship’s ballast water. It was necessary to test the detection of bacteria while which such sedimentary materiel was simulated in experimental solutions. Still, it is a better simultaneous detection of bacteria, microalgae and standard micro-particles on one chip. So that the average size of 2 µm of *C. volutis* microalgae and 1 µm size of standard polystyrene fluorescence particles were added into the test tube which had *Enterococci* and *E. coli*. The mixed sample solution was carefully cultured with the protocols and a filtration method used to ensure that the ingredient sizes were not to be more than 2 µm. The optical image of mixed sample solution was taken by the microscope and it was shown in Figure 8. The system even proved valid for specific testing, but there were some problems encountered in actual detection experiments for bacteria, microalgae and particles. One reason was size differences. A further reason was the nature and characteristic of living organisms. The particles used in the experiment were quite different from living organisms. Nevertheless, impedance pulse detection based on samples’ size and volume was a favorable option in starting detection. Therefore, the problems were troubleshooting and also the pre-amplifier circuit, filter amplifier circuit, and signal processing circuit for RPS detection were developed. Finally, the results for simultaneous detection of the mixed solution were successfully produced as in the following Figure 9. Among the four kinds of samples, it was clearly seen that all standard 1 µm size particles were detected at the same amplitude (here they were shown by pink ellipses in the picture), while others have the range of amplitudes which depends on their various sizes. Finally, we counted the total number of signals for individual samples after a ten minute duration. These statistics were used to develop the diagram shown in Figure 10. The histogram indicates that the smallest size samples flow faster in the microchannel and the level of detection was dependent on the level of the sample concentration.

## 5. Conclusions

As targeted to comply with the standard D-2 of international convention on the management of ship’s ballast water and sediment, label-free various microalgae, and bacteria samples, such as *Chlorella volutis*, *Dunaliella salina*, *Platymonas subcordiformis*, *Chrysophytes*, *Escherichia coli*, *Enterococci*, and polystyrene particles, were successfully detected and analyzed by using a changeable lab-on-a-chip detector which was based on impedance pulse sensing and chlorophyll fluorescence intensity detection. When compared with the existing detection technology, microfluidic chip platform technology has parallel advantages in the detection processes: it is a simple and easy to operate; it provides on board automatic complete detection process in a very short time; it is of a small size; it is fast but high sensitivity is achieved in detection; and, gives good accurate in results. The advantages of integrating LED light induced chlorophyll fluorescence method and microfluidic chip technology are that they can realize not only continuous detection on chlorophyll content of single cell microalgae, but also their activity in real time situation. The detector has a tolerance on influence factors of pH value, temperature, salinity, and others interference factors in sample solution due to the fact that the micron scale samples solution volume are needed to complete the whole operation of detection. It is thus has highly suitable for in-situ uses. The changeable lab-on-a-chip detector can also be provided a portable on-line system of analysis for micro-organisms by applying detection software program and the display devices on the structure.

## References

[1] Katsanevakis S., Wallentinus I., Zenetos A., Leppäkoski E., Çinar M.E., Oztürk B., Grabowski M., Golani D., Cardoso C. (2014). Impacts of invasive alien marine species on ecosystem services and biodiversity: A pan-European review. Aquat. Invasions.

[2] Dupont R.R., Ganesan K., Theodore L. (2016). Pollution Prevention: Sustainability, Industrial Ecology, and Green Engineering.

[3] Gollasch S., David M., Voigt M., Dragsund E., Hewitt C., Fukuyo Y. (2007). Critical review of the IMO international convention on the management of ships’ ballast water and sediments. Harmful Algae.

[4] World Maritime News Ballast Water Management Convention. http://worldmaritimenews.com/archives/229362/ballast-water-management-convention-enters-into-force/.

[5] Lagersmit: The Origin of Sealing Solutions. https://www.lagersmit.com/update-ballast-water-management-convention/.

[6] IMO International Maritime Organization. http://www.imo.org/en/About/Conventions/ListOfConventions/Pages/International-Convention-for-the-Control-and-Management-of-Ships’-Ballast-Water-and-Sediments-(BWM).aspx.

[7] Washington J.A., White C.M., Laganiere R.N.M., Smith L.H. (2016). Detection of significant bacteriuria by microscopic examination of urine. Lab. Med..

[8] Alsteens D., Trabelsi H., Soumillion P., Dufrêne Y.F. (2013). Multiparametric atomic force microscopy imaging of single bacteriophages extruding from living bacteria. Nat. Commun..

[9] Pol E., Coumans F.A.W., Grootemaat A.E., Gardiner C., Sargent I.L., Harrison P., Sturk A., Leeuwen T.G., Nieuwland R. (2014). Particle size distribution of exosomes and microvesicles determined by transmission electron microscopy, flow cytometry, nanoparticle tracking analysis, and resistive pulse sensing. J. Thromb. Haemost..

[10] News Medical Life Sciences. https://www.news-medical.net/life-sciences/How-Does-a-Coulter-Counter-Work.aspx.

[11] Yu A.C., Loo J.F., Yu S., Kong S.K., Chan T.F. (2014). Monitoring bacterial growth using tunable resistive pulse sensing with a pore-based technique. Appl. Microbiol. Biotechnol..

[12] Qin Z., Zhe J., Wang G.X. (2011). Effects of particle’s off-axis position, shape, orientation and entry position on resistance changes of micro Coulter counting devices. Meas. Sci. Technol..

[13] Zhe J., Jagtiani A., Dutta P., Hu J., Carletta J. (2007). A micromachined high throughput Coulter counters for bioparticle detection and counting. J. Micromech. Microeng..

[14] Puchkov E. (2016). Image analysis in microbiology: A review. J. Comput. Commun..

[15] Wiedemann P., Schneider F.K., Suhr H. (2016). Image processing for identification and quantification of filamentous bacteria in in situ acquired images. Biomed. Eng. Online.

[16] Pospichalova V., Svoboda J., Dave Z., Kotrbova A., Kaiser K., Klemova D., Ilkovics L., Hampl A., Crha I., Jandakova E. (2015). Simplified protocol for flow cytometry analysis of fluorescently labeled exosomes and microvesicles using dedicated flow cytometer. J. Extracell. Vesicles.

[17] Viswanath D.I., Mace E.M., Hsu H.T., Orange J.S. (2017). Quantification of natural killer cell polarization and visualization of synaptic granule externalization by imaging flow cytometry. Clin. Immunol..

[18] Shapiro H.M. (2005). Practical Flow Cytometry.

[19] Wang J., Maw M.M., Yu X., Dai B., Wang G., Jiang Z. (2017). Applications and perspectives on microfluidic technologies in ships and marine engineering: A review. Microfluid. Nanofluid..

[20] Zhang J., Yan S., Yuan D., Alici G., Nguyen N.T., Warkiani M.E., Li W. (2016). Fundamentals and applications of inertial microfluidics: A review. Lab Chip.

[21] Stone H.A., Kim S. (2001). Microfluidics: Basic issues, applications, and challenges. AIChE J..

[22] Elvira K.S., Solvas X.C., Wootton R.C. (2013). The past, present and potential for microfluidic reactor technology in chemical synthesis. Nat. Chem..

[23] Wang J., Song Y., Maw M.M., Song Y., Pan X., Sun Y., Li D. (2015). Detection of size spectrum of microalgae cells in an integrated underwater microfluidic device. J. Exp. Mar. Biol. Ecol..

[24] Smith G.D., Takayama S. (2017). Application of microfluidic technologies to human assisted reproduction. Mol. Hum. Reprod..

[25] Zhang F., Dan G.A.O., Liang Q.L. (2016). Advances of microfluidic technologies applied in bio-analytical chemistry. Chin. J. Anal. Chem..

[26] Maw M.M., Wang J., Li F., Jiang J., Song Y., Pan X. (2015). Novel electrokinetic microfluidic detector for evaluating effectiveness of microalgae disinfection in ship ballast water. Int. J. Mol. Sci..

[27] Avilés C.P., Feliu E.J., Villagrasa J.P., del Zamora B.M., Corbera A.H., Farrarons J.C., Català P.L.M., Samitier J. (2016). Combined dielectrophoresis and impedance systems for bacteria analysis in microfluidic on-chip platforms. Sensors.

[28] Anderson W., Lane R., Korbie D., Trau M. (2015). Observations of tunable resistive pulse sensing for exosome analysis: Improving system sensitivity and stability. Langmuir.

[29] Yang L., Yamamoto T. (2016). Quantification of virus particles using nanopore-based resistive-pulse sensing techniques. Front. Microbiol..

[30] Du L., Zhe J., Carletta J., Veillette R., Choy F. (2010). Real-time monitoring of wear debris in lubrication oil using a microfluidic inductive Coulter counting device. Microfluid. Nanofluid..

[31] Carminati M. (2017). Advances in high-resolution microscale impedance Sensors. J. Sens..

[32] Heo J., Hua S.Z. (2009). An overview of recent strategies in pathogen sensing. Sensors.

[33] Kozak D., Anderson W., Vogel R., Trau M. (2011). Advances in Resistive Pulse Sensors: Devices bridging the void between molecular and microscopic detection. Nano Today.

[34] Wang J., Zhao J., Wang Y., Wang W., Gao Y., Xu R., Zhao W. (2016). A new microfluidic device for classification of microalgae cells based on simultaneous analysis of chlorophyll fluorescence, side light scattering, resistance pulse sensing. Micromachines.

[35] Constantinescu C. (2003). Trends and challenges in VLSI circuit reliability. IEEE Micro.

[36] Han S.Y., Kim B.R., Ko H.Y., Kwon H.K., Kim B.I. (2016). Assessing the use of quantitative light-induced fluorescence-digital as a clinical plaque assessment. Photodiagn. Photodyn. Ther..

[37] Kühnisch J., Weltzien H.R. (2004). Quantitative light-induced fluorescence (GLF)-a literature review. Int. J. Comput. Dent..

[38] Pretty I.A., Edgar W.M., Higham S.M. (2003). A review of the effectiveness of quantitative light-induced fluorescence (QLF) to detect early caries. Sci. Am..

[39] Huo D.Q., Liu Z., Hou C.J., Yang J., Luo X.G., Fa H.B., Dong J.L., Zhang Y.C., Zhang G.P., Li J.J. (2010). Recent advances on optical detection methods and techniques for cell-based microfluidic systems. Chin. J. Anal. Chem..

[40] Ferreira Zandoná A., Ando M., Gomez G.F., Garcia-Corretjer M., Eckert G.J., Santiago E., Katz B.P., Zero D.T. (2013). Longitudinal analyses of early lesions by fluorescence: An observational study. J. Dent. Res..

[41] Encyclopedia.com. http://www.encyclopedia.com/plants-and-animals/microbes-algae-and-fungi/moneran-and-protistan/algae.

[42] Hosikian A., Lim S., Halim R., Danquah M.K. (2010). Chlorophyll extraction from microalgae: A review on the process engineering aspects. Int. J. Chem. Eng..

